# Odontogenic Brain Abscess in a Hereditary Haemorrhagic Telangiectasia (HHT) Patient: Case Report with a Comprehensive Literature Review

**DOI:** 10.3390/tropicalmed11030067

**Published:** 2026-03-02

**Authors:** Pontus Westerström, Joanna Malgorzata Bivand, Øyvind Kommedal, Birgitta Ehrnström, Joakim Stray Andreassen, Jan Egil Afset

**Affiliations:** 1 Department of Medical Microbiology, Clinic of Laboratory Medicine, St. Olavs Hospital, Trondheim University Hospital, 7006 Trondheim, Norway; 2 Department of Microbiology, Haukeland University Hospital, 5021 Bergen, Norway; 3 Department of Clinical Science, University of Bergen, 5021 Bergen, Norway; 4 Department of Infectious Diseases, Clinic of Medicine, St. Olavs Hospital, Trondheim University Hospital, 7006 Trondheim, Norway; 5 Department of Clinical and Molecular Medicine, Faculty of Medicine and Health Sciences, Norwegian University of Science and Technology, 7006 Trondheim, Norway; 6 Department of Neurosurgery, Neuroclinic, St. Olavs Hospital, Trondheim University Hospital, 7006 Trondheim, Norway; 7 Department of Circulation and Medical Imaging, Faculty of Medicine and Health Sciences, Norwegian University of Science and Technology, 7006 Trondheim, Norway

**Keywords:** odontogenic brain abscess, oral microbiome, *Capnocytophaga*, *Campylobacter gracilis*, *Arachnia propionica*, Candidatus Saccharibacteria oral taxon 488, 16S rRNA gene sequencing, next-generation sequencing, case report

## Abstract

Background: Recent diagnostic methods have enabled the detection of often culture-negative pathogens, including anaerobic bacteria from the oral cavity. Characterising the microbial diversity and co-occurrence of bacteria in such infections is important for understanding the molecular pathophysiology in odontogenic brain abscesses. Case presentation: We describe a case of polymicrobial odontogenic brain abscess in a 59-year-old man of Vietnamese ethnicity with a documented increased risk of brain abscess due to Hereditary Haemorrhagic Telangiectasia (HHT). The microbiological diagnostic work-up included conventional culture, matrix-assisted laser desorption ionization-time of flight mass spectrometry (MALDI-TOF MS), targeted 16S rDNA analysis using three broad-range group-specific PCR (polymerase chain reaction) assays and next-generation sequencing (NGS). A literature review was conducted, including database searches for each identified microorganism. Twelve anaerobic bacterial species were detected, among which *Treponema medium*, *Capnocytophaga* HMT-323 and *Candidatus Saccharibacteria* oral taxon 488 have not previously been reported in brain abscesses. In addition, we identified the extremely rare pathogens *Arachnia propionica* and *Capnocytophaga ochracea*. Conclusion: This is the first report of *Ca. Saccharibacteria* oral taxon 488 in a clinical sample and the first detection of any species from this phylum in a brain abscess, co-detected with *A. propionica,* consistent with its obligate epibiotic lifestyle. Our findings broaden the known microbial diversity associated with odontogenic brain abscesses and underscore the value of 16S rDNA NGS in characterising polymicrobial infections, particularly when fastidious or uncultivable organisms are involved.

## 1. Introduction

Brain abscesses are rare infections with an incidence of about 0.4–0.9 per 100,000 population [1,2]. They occur more often in patients with Hereditary Haemorrhagic Telangiectasia (HHT), also known as Osler–Weber–Rendu disease [3,4]. HHT is an autosomal dominant hereditary condition diagnosed in accordance with the international Curaçao criteria by epistaxis, mucocutaneous telangiectasias and arteriovenous malformations in the lungs, abdomen or brain [3,4,5]. The pulmonary arteriovenous malformations (PAVMs) are present in over 20% of HHT patients, but arteriovenous malformations can also present in other anatomical locations, such as the liver and the central nervous system. PAVMs predispose to mainly neurological complications, such as stroke and cerebral abscesses, but abscesses also present outside the central nervous system. The pathophysiological mechanism is explained by impaired capillary filtering (right-to-left shunts), providing microbiological pathogens a passage to the arterial circulation [6]. Brain abscesses in HHT patients are typically polymicrobial [3,7,8].

The prevalence of HHT varies from 75 per 100,000 in the Dutch Antilles, 42 per 100,000 population in the French region Ain, to 2.5 per 100,000 in northern England [9,10]. The increased risk of brain abscesses in people with pulmonary right-to-left shunting was described by Reading in 1932 and first reported in a HHT patient in 1951 [11,12]. In Denmark, where the prevalence of HHT is 15 per 100,000, the incidence of brain abscesses in the HHT population (7.8% among HHT with PAVM) has been calculated to be over 100-fold increased as compared to the general population [4]. However, more recent data suggest a more modest increased risk (20-fold), and data from the USA have estimated a risk ratio closer to 40-fold [13,14]. Over the last four decades, the introduction of magnetic imaging and advanced surgical techniques have contributed to decreased mortality in patients with brain abscesses, from 30–70% to 10–15%, while the mortality rate in the subset of patients with HHT has been reported to be about 40%, although updated statistics are not available [15,16,17].

## 2. Case Presentation

**Figure 1:**
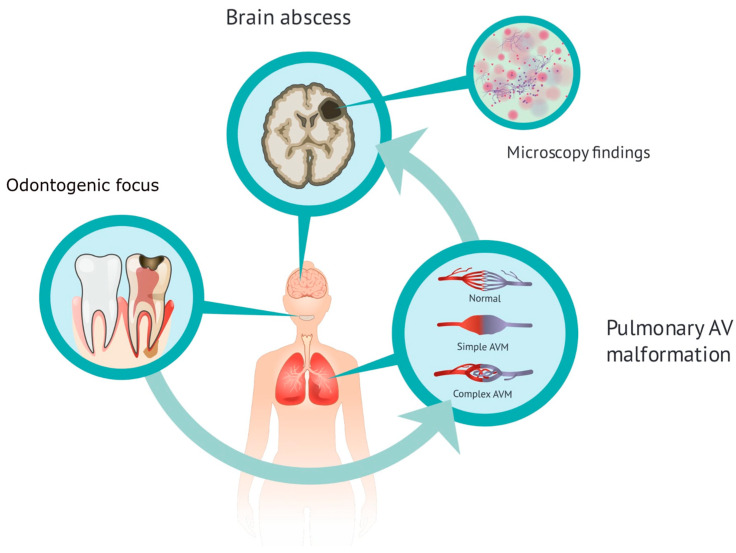
Illustration of the pathophysiology of an odontogenic brain abscess in the HHT patient.

**Figure 2:**
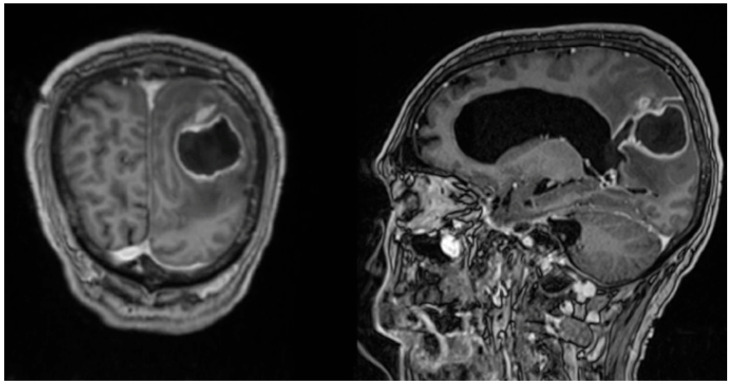
Magnetic Resonance Image of the brain showing left-side parietooccipital abscess in the study patient: horizontal view (**left**) and lateral view (**right**).

**Figure 3:**
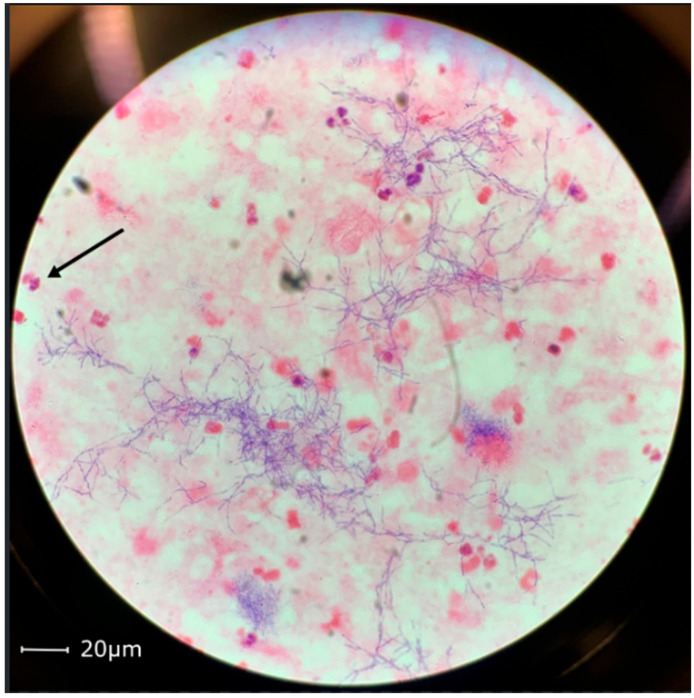
Gram-positive branching rods in brain abscess material from the case study patient. Scale bar: 20 µm. The black arrow indicates a neutrophil granulocyte with an average diameter size of 12–15 µm.

This case demonstrates a 59-year-old male patient of Vietnamese ethnicity with HHT, who developed brain abscess twice, the second time with fatal outcome. The patient was first diagnosed with HHT in 2006. He suffered from recurrent epistaxis, and gastric angiodysplasia was observed during gastroscopy. Right-side pulmonary arteriovenous malformation and a spleen aneurysm were described on a computed tomography of the thorax and abdomen. Family history was unavailable at the time. In 2012, the patient was successfully treated at St. Olavs Hospital (Trondheim, Norway) for a thalamic abscess with neurosurgery and antibiotics. Pus culture showed growth of *Fusobacterium nucleatum*. It was considered that the likely cause of infection was the patient’s poor dental status (Figure 1). In 2021, the patient was diagnosed with epilepsy and was started on prophylactic treatment with Levetiracetam. He was again admitted to the Emergency Department at St. Olavs Hospital in November 2022 with epileptic seizures and fever. A computed tomography revealed a mass measuring 26 × 46 mm^2^ in the left parietooccipital region with findings indicating a brain abscess (Figure 2). Acute neurosurgical drainage was performed, and direct microscopy of the pus demonstrated Gram-positive branching rods (Figure 3). The microbiology resident suspected *Actinomyces* or *Nocardia*. Three days after diagnosis, the abscess breached into the neighbouring left lateral ventricle and an external ventricular drain was introduced for additional intrathecal treatment with amikacin. The pus sample was cultivated on agar plates, and after six days of incubation, growth of *Arachnia propionica* was observed. Moreover, *Capnocytophaga ochracea* was detected and identified by amplification and Sanger sequencing of the 16S rRNA gene. Additionally, the pus sample was sent to the Department of Microbiology at Haukeland University Hospital in Bergen for extended 16S sequencing [18]. The extended analysis identified three additional bacterial species, *Fusobacterium nucleatum*, *Campylobacter gracilis* and *Treponema medium,* and confirmed the presence of *A. propionica* and *Campylobacter* sp.

**Figure 4:**
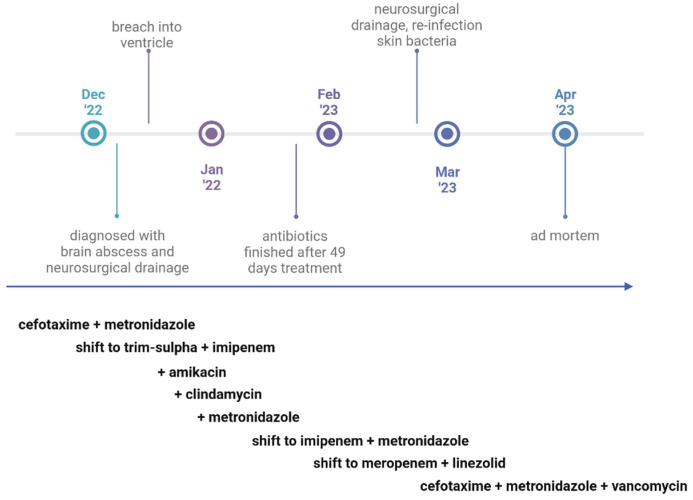
Timeline of antimicrobial treatment. The microbiological guidance for the chosen antibiotics is explained under the Section 2.

The brain abscess was first treated with intravenous cefotaxime and metronidazole, which was shifted to intravenous imipenem/cilastatin and trimethoprim-sulfamethoxazole when Gram-positive branching filamentous rods were observed by Gram stain microscopy, with the addition of 14 days of intrathecal amikacin when ventriculitis was detected (Figure 4). Upon identification of *A. propionica* and *C. ochracea,* intravenous metronidazole and intravenous clindamycin were added to the treatment. The treatment was then shifted to imipenem and metronidazole, but due to the high imipenem Mean Inhibitory Concentration (MIC) value of the *Capnocytophaga* isolate, it was changed to meropenem (with a lower MIC than imipenem) and linezolid. During the first treatment period, the patient received antibiotics for 49 days. The patient was transferred to a rehabilitation unit, but after four weeks, he deteriorated cognitively and was readmitted to the hospital. The abscess size had increased, and he was reoperated on with neurosurgical drainage. Three new bacterial species were found after cultivation of pus from the abscess: *Staphylococcus aureus*, *Staphylococcus epidermidis* and *Staphylococcus capitis*. This time, the patient did not respond to treatment with relevant antibiotics (intravenous cefotaxime, metronidazole and vancomycin). Because the patient’s clinical condition was considered too poor for additional surgery and active treatment, further interventions were withheld. He was transferred to palliative care, where he died four months after the brain abscess was diagnosed.

Gram-positive branching filamentous rods in the brain abscess aspirate (Figure 3) were identified by direct microscopy. Abscess material was cultured on microbiological agar plates under aerobic and anaerobic conditions in accordance with standard operating procedures at the laboratory. After four days, there was growth identified by matrix-assisted laser desorption/ionisation time-of-flight mass spectrometry (MALDI-TOF MS) using MALDI Biotyper Microflex software version 11.0.0.0_9607-10833 (RUO) with MBT Compass Library DB-10833 Mass Specter Profile (Bruker Daltonics, Mannheim, Germany) as *C. ochracea* on agar media incubated aerobically. After six days of incubation under anaerobic conditions, growth of morphologically different colonies was observed, and a second bacterial species was identified by MALDI-TOF MS as *A. propionica*. The pus samples were incubated under aerobic and anaerobic conditions for 14 days and 10 days, respectively.

Residual sample material was sent overnight with cooling to Haukeland University Hospital for an extended targeted analysis of the pus sample using a set of three broad-range group-specific 16S rDNA (ribosomal DNA) PCRs followed by Sanger sequencing and interpretation of chromatograms using the application RipSeq Mixed version 10.0 (Pathogenomix Inc., Santa Cruz, CA, USA) [18]. This analysis identified *A. propionica*, *T. medium*, *C. gracilis*, *F. nucleatum* and *Capnocytophaga* sp.

In addition, the collected pus sample was post-mortem analysed using 16S next-generation sequencing (NGS) at the Department of Microbiology, Haukeland University Hospital, on the Illumina MiSeq system (Illumina, Redwood City, CA, USA). The Illumina protocol for 16S deep sequencing was followed with some modifications as described previously [19,20]. Sample material and/or extracted bacterial DNA were stored at −80 °C until sequencing. Negative and positive sample process controls were included. Sequencing data was analysed using the RipSeq NGS software version 10.0 (Pathogenomix Inc). For taxonomic assignments, CLSI (Clinical & Laboratory Standards Institute) guidelines were followed; for valid species identification, ≥99% homology with a high-quality reference and a minimum distance of >0.8% to the next alternative species. Sequences representing contaminant bacterial DNA were filtered as described by Dyrhovden et al. [21,22] (Table 1).

The 16S NGS analysis identified *A. propionica*, *T. medium*, *C. gracilis*, *F. nucleatum*, *Capnocytophaga* sp. HMT-323, *Treponema maltophilum*, *Schaalia georgiae*, *Treponema socranskii*, *Candidatus Saccharibacteria* oral taxon 488, *Tannerella forsythia* and *Johnsonella ignava* (Table 2).

Susceptibility testing with MIC Gradient Test (Liofilchem, Roseto degli Abruzzi, Italy) was performed on Brucella Agar. MIC results were reported without S-I-R classification since clinical breakpoints have not been established for these bacterial species by European Committee on Antimicrobial Susceptibility Testing (EUCAST). However, in cases where MIC values were higher than the Epidemiological Cut-off value (ECOFF) of a bacterial species, the isolate was classified as resistant to that antibiotic drug. *C. ochracea* was incubated in 5% CO_2_ atmosphere at 35 ± 2 °C. MIC values (mg/L) after 44 h of incubation were: ampicillin 0.125, cefotaxime 0.032, ciprofloxacin 0.016, gentamicin 128 (resistant), imipenem 0.5, meropenem 0.032 and benzylpenicillin 0.125. MICs (mg/L) of *A. propionica* after 76 h anaerobic incubation (Whitley A45 anaerobic workstation, Don Whitley Scientific, Shipley, UK) were ampicillin 0.064, cefotaxime 0.125, meropenem 0.032, benzylpenicillin 0.032, piperacillin-tazocin 0.064 (read after 24 h), clindamycin 0.5 (read after 24 h), and vancomycin 1.0. The isolate was categorised as resistant to metronidazole based on results from the disk diffusion test.

## 3. Literature Review

We identified relevant publications by database searches on (microbe name) AND (brain abscess AND cerebral abscess) in PubMed and Google Scholar. Additional publications were identified from reference lists. We limited inclusion to publications written in English until the end of 2023.

The term polymicrobial is often used to describe infections with two or more bacterial species, and we have applied this criterion in this study.

### 3.1. Arachnia propionica

*A. propionica* is an anaerobic Gram-positive pleomorphic rod, which belongs to the normal oral microbiome. *A. propionica* was first mistaken as *Actinomyces israelii* and has been referred to in the literature under four different names (*Actinomyces propionicus*, *Arachnia propionica*, *Propionibacterium propionicus and Pseudopropionibacterium propionicum*) [45,46,47,48,49,50]. *A. propionica* is reported to be associated with infections in different anatomical sites (lacrimal glands, abdomen, lung), but has only been described twice in brain abscesses. Two male patients aged 32 and 33, both suffering from Eisenmenger syndrome, with right-to-left shunting of non-capillary filtered blood to the central nervous system circulation, were diagnosed with occipital and frontal brain abscess, respectively [43,44].

### 3.2. Capnocytophaga ochracea

*C. ochracea* is a Gram-negative, facultative anaerobic, capnophilic, rod-shaped bacterial species in the *Flavobacteriaceae* family, with a genus consisting of 22 species, which all may be found as part of the human oral microbiome [51]. The *Capnocytophaga* that colonise dogs and cats include *C. canimorsus* (first described as Dysgonic fermenter 2), *C. canis*, *C. cynodegmi* and *C. felis* [52,53]. To our knowledge, *Capnocytophaga* spp. has been reported twelve times as the cause of brain abscess, but only one of these was *C. ochracea* [23,24,25,26,27,28,29,30,31,32,33,34,35]. The *C. ochracea* case was a 7-year-old boy with prior tooth extraction and a frontal abscess with multi-resistant *C. ochracea* [28]. In addition, another six cases of *Capnocytophaga* spp. were identified in cohort studies of brain abscesses using metagenomic 16S analysis [31,32,54].

### 3.3. Fusobacterium nucleatum

*F. nucleatum* is an anaerobic Gram-negative rod, which can be found in the human microbiome. The genus *Fusobacterium* currently includes about 35 named taxa, with 16 valid species [55]. *Fusobacterium necrophorum*, known to cause Lemierre’s syndrome, and *F. nucleatum* are the two species most commonly seen in human infections. There is a documented co-occurrence of *F. nucleatum* in brain abscess and periodontal disease. *F. nucleatum* is isolated from 2% of brain abscesses, and it has been suggested that it grows synergistically with other pathogens [56,57].

With the availability of more advanced diagnostic methods, it is likely that the detected prevalence will increase.

### 3.4. Capnocytophaga *HMT-323*

*C*. HMT-323 is part of the human oral microbiome [58]. To our knowledge, *C*. HMT-323 has never been reported from a brain abscess before.

### 3.5. Campylobacter gracilis

*C. gracilis* is an anaerobic Gram-negative rod in the human oral microbiome, known to cause periodontal and endodontal infections, but has also been reported in other severe infections such as pulmonary empyema and bacteraemia [36,59,60,61,62]. We have identified four publications which report on *C. gracilis* from brain abscesses. The first case, from 1990, is very similar to the case in this report. A 57-year-old woman was diagnosed with HHT, pulmonary arteriovenous fistulas and a thalamic lesion. Cultivation of pus from the lesion resulted in growth of many anaerobic oral pathogens, among them both *F. nucleatum* and *C. gracilis* [37]. The second report describes a patient with an odontogenic brain abscess, caused by *C. gracilis* and *F. nucleatum* [41]. The third case describes a 35-year-old pregnant woman who had a tooth extraction in the 20th gestational week. She received a short course of oral ampicillin and two weeks later developed a subdural empyema. *Bacteroides fragilis*, *Wolinella* spp., *C. gracilis* and *Prevotella buccae* [39] grew in pus aspirated by neurosurgical drainage. The fourth case also represents a 35-year-old woman, who post-partum developed a grand mal seizure and was diagnosed with a parietal mass. Aerobic cultivation of pus from the brain abscess resulted in the growth of *Streptococcus constellatus*, Gram-negative rods, and anaerobic Gram-negative cocci were found in anaerobic cultures. The Gram-negative rods were identified as *C. gracilis* by 16S rRNA gene sequencing [38]. In addition, *C. gracilis* has been identified by 16S rRNA gene metagenomic studies of brain abscesses in 22 cases [18,31,32,40,42,63].

### 3.6. Treponema medium

*T. medium* is an anaerobic spirochete, first described in 1997 by Umemoto et al., isolated from a patient with periodontal disease [64]. There are 28 validly published *Treponema* species, pathogenic and non-pathogenic to humans [65]. The most widely known infection caused by bacteria of this species is the sexually transmitted disease syphilis (*T. pallidum* subsp. *pallidum*), yaws (*T. pallidum* subsp. *pertenue*), endemic syphilis (*T. pallidum* subsp. *endemicum*) and pinta (*T. carateum*) [66]. However, at least 50 different sequenced *Treponema* phylotypes are part of the oral microbiome, and many of these are associated with periodontal and gingival disease [67,68]. To our knowledge, *T. medium* has not previously been reported from a brain abscess.

### 3.7. Treponema maltophilum

*T. maltophilum* is an anaerobic spirochete associated with periodontal lesions [69]. It has been reported on at least one occasion from a patient with a brain abscess in a metagenomic study [18].

### 3.8. Schaalia georgiae

*S. georgiae* is an anaerobic, Gram-positive, rod-shaped bacterium found in the human periodontal flora, first described by Johnson et al. in 1990, formerly known as *Actinomyces georgiae* [70]. There are currently 50 known species of the genus *Actinomyces*, and they are known to cause mainly opportunistic human infections with typical granulomatous histopathology, which defines the disease [71]. In a publication with massive parallel sequencing, two cases of *S. georgiae* were reported in brain abscesses [45].

### 3.9. Treponema socranskii

*T. socranskii* is an anaerobic spirochete first described in 1984 by Smibert et al. in patients with periodontitis [72]. It has been reported from a brain abscess in one previous publication [73].

### 3.10. Candidatus Saccharibacteria Oral Taxon 488

The phylum *Candidatus Saccharibacteria* (formerly TM7) is part of the oral microbiome [74]. *Ca. Saccharibacteria* are ultrasmall parasitic bacteria (200 to 300 nm) found on the surface of their bacterial hosts [75]. The first cultivation of a *Ca. Saccharibacteria* epibiont bacteria-parasite (Nanosynbacter lyticus type strain TM7x; HMT-952) was performed by He et al. in 2015 with the host *Actinomyces odontolyticus actinosynbacter* strain XH001 [76]. More recently, it has been reported that *Ca. Saccharibacteria* oral taxon 488 has been isolated in co-culture with *A. propionica*, which supports the co-occurrence of the two species reported in this study [77]. To our knowledge, this is the first clinical case reporting *Ca. Saccharibacteria* oral taxon 488, and no other species from the phylum *Ca. Saccharibacteria* has previously been detected in a brain abscess. *Ca. Saccharibacteria* are grouped into the periodontal pathogenic “red complex” (explained in Section 3.11), making them likely to be “inflammophilic” bacteria that prefer a nutrition-rich environment [78].

### 3.11. Tannerella forsythia

*T. forsythia* was first described in 1986 as *Bacteroides forsythus* by Tanner et al. [79]. It is an anaerobic Gram-negative bacterium involved in periodontal disease, which belongs to the pathogenic “red complex” (the two other members of the red complex group are *Porphyromonas gingivalis* and *Treponema denticola*) [80]. Socransky et al. defined five bacterial complexes (red, orange, purple, yellow, and green) in the subgingival biofilm with different levels of pathogenicity, where the green, yellow and purple complexes represent early colonisers and the orange and red complexes represent the more pathogenic late colonisers associated with chronic periodontitis. *T. forsythia* was reported in two brain abscesses in a massive parallel sequencing study [45,78,81].

### 3.12. Johnsonella ignava

*J. ignava* was first described by Moore and Moore in 1994 as an anaerobic Gram-negative rod from the human gingival crevice [82]. It was reported in a study with massive parallel sequencing in an odontogenic brain abscess [45].

## 4. Discussion

Over the past 15 years, microbial pathogen identification has undergone considerable improvement with the development of more advanced diagnostic methods, including sequencing technologies. The introduction of MALDI-TOF MS has considerably reduced the time for reporting microbial identification from bacterial cultures. This enables earlier targeted antimicrobial treatment based on bacterial species. However, in complex infections, such as brain abscesses, which are often polymicrobial and contain anaerobic bacteria, identification of pathogens by culture only is still challenging. 16S sequencing increases the ability to detect a wider range of pathogens present in a sample, including those normally difficult to cultivate (such as fastidious and anaerobic bacteria). In this report, we present unique brain abscess findings of *T. medium*, *Capnocytophaga* sp. HMT-323 and *Ca. Saccharibacteria* oral taxon 488. In addition, we report the extremely rare findings of *T. socranskii* and *A. propionica* in a brain abscess. However, the identification of *C. ochracea* by MALDI-TOF MS, but not by NGS, may suggest the possibility that *Capnocytophaga* sp. HMT-323 was misidentified as *C. ochracea.* MALDI-TOF MS is generally good at discriminating between different *Capnocytophaga* species, but as of today, *Capnocytophaga* sp. HMT-323 is not included in the MALDI-TOF MS database [83]. Other researchers have previously proved 16S NGS analysis to be a powerful tool for detecting numerous fastidious microbial pathogens in samples from brain abscesses [18,46].

PAVM and cardiac conditions with right-to-left shunting, dental disease (caries, inflammation, infection or recent tooth extraction) and sinusitis have been reported in many patients who have developed anaerobic brain abscesses (Table 3).

HHT patients are frequently diagnosed with PAVM during a first-time episode with a brain abscess. It has been recommended that the PAVM should be treated with embolisation to prevent future brain abscesses [4]. Our case illustrates the importance of this, since plans to remove the pulmonary malformations were initiated only after the patient presented with the second and fatal brain abscess. Our literature review findings also support the established association of *C. canimorsus* infection with recent exposure to saliva from dogs and cats [84] (Table 3). In addition, there is an increased risk of developing an anaerobic brain abscess in patients which are immunocompromised with various conditions, such as Tumor-Necrosis-Factor alpha (TNF-α) treatment, diabetes, alcoholism, pregnancy and post-partum condition (Table 3).

**Figure 5:**
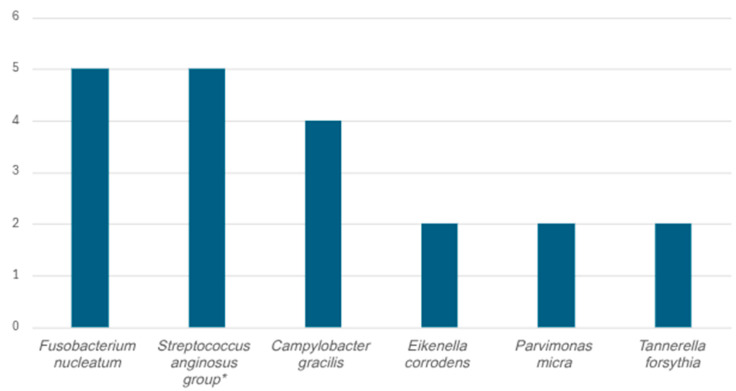
Co-detection in brain abscesses with *Capnocytophaga* spp. (*n* = 17) of different anaerobic bacteria as reported in published literature (Table 1). * Streptococcus anginosus group includes *S. anginosus*, *S. intermedius* and *S. constellatus*.

The oral microbiome has gained focus with its about 800 bacterial species being increasingly linked to invasive diseases such as bacteraemia, pleural infection and brain abscess [47,85]. The mainly polybacterial odontogenic brain abscesses are composed of several families of microorganisms; 5–12% of all brain abscesses are considered to be of odontogenic origin [86,87]. In a recent publication, Hsu et al. list 81 genera of bacteria associated with brain abscesses, many of them bacteria which are part of the oral microbiome [88]. The findings in the patient in our study support both the considerable diversity and the most frequently detected microbes reported in such abscesses. The classification of a brain abscess as of odontogenic origin is traditionally made by the exclusion of other foci, and therefore, the calculated proportion of brain abscesses of odontogenic origin may be imprecise [89]. In studies of odontogenic brain abscesses with *Capnocytophaga* spp. and *C. gracilis* findings in 16S NGS analysis (including our findings), there have been an average of 8 (range 5–12, median 8) detected bacterial species [18,45,46,47,62]. In polymicrobial brain abscesses with *Capnocytophaga* spp., it is most often co-detected with *F. nucleatum* and the *Streptococcus anginosus* group (Figure 5).

**Figure 6:**
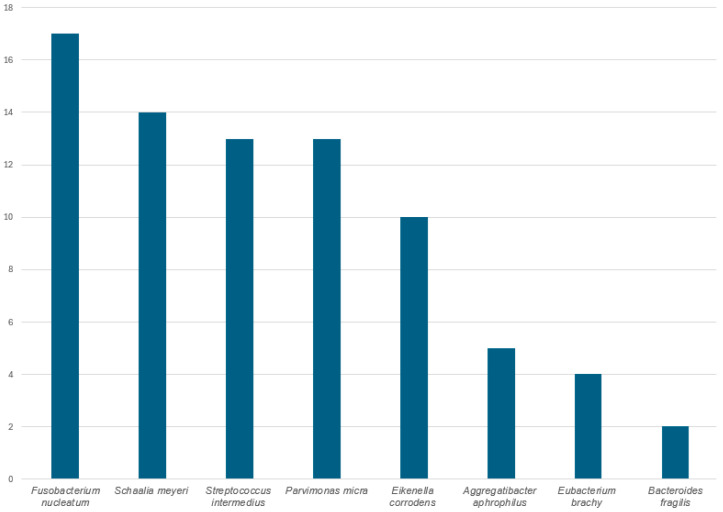
Co-detection in brain abscesses with *Campylobacter gracilis* (*n* = 22) of different anaerobic bacteria as reported in published literature (Table 1).

In polymicrobial brain abscesses with *C. gracilis,* the bacterium is most commonly co-detected with *F. nucleatum*, *Schaalia meyeri* and *S. intermedius* (member of *Streptococcus anginosus* group) (Figure 6)*,* similar to what other authors have reported [45,60,86].

A recent study of 44 patients with odontogenic brain abscesses reported a high prevalence of the *S. anginosus* group (88%) compared to a non-odontogenic brain abscess group (7%) [90]. A Norwegian study of 37 spontaneous (non-post-operative or traumatic) brain abscesses found that *Streptococcus intermedius*, *F. nucleatum* and *Aggregatibacter aphrophilus* were often identified in brain abscesses of assumed oral or sinus origin [45]. It was suggested that these three bacterial species, alone or together, act as pioneer pathogens (early colonisers) in an oxygenated zone such as the brain and prepare conditions for strict anaerobic representatives of the oral microbiome (late colonisers) [46]. Pulp infections are reportedly associated with *F. nucleatum* in co-occurrence with *C. gracilis* (and many other bacteria) [91]. Possibly, the oral microbiome interactions in a brain abscess have similar pathophysiological mechanisms as those in an odontogenic infection, but mapping of clusters and co-occurrence of microbiological pathogens in odontogenic brain abscesses is yet to be better understood.

To strengthen diagnostics in odontogenic brain abscesses, we support the suggestion of introducing a routine oral surgeon consultation in patients with suspected odontogenic brain abscess, similar to having infectious endocarditis patients examined for oral infectious foci. Also, it is important to match microbiological results from intracranial and extracranial sites to verify an odontogenic origin [92].

We present unique findings of *T. medium*, *Capnocytophaga* sp. HMT-323 and *Ca. Saccharibacteria* oral taxon 488 in co-occurrence with nine other bacteria in an odontogenic brain abscess from a HHT patient with poor dental status. In this report, we highlight the complex nature of co-aggregation of bacteria in odontogenic brain abscesses. We wish to address the interesting co-detection of *A. propionica* and *Ca. Saccharibacteria* oral taxon 488, the latter, to our knowledge, being the first ever reported detection in a clinical sample. The phylum *Candidatus Saccharibacteria* has been detected in clinical specimens (blood cultures and cardiac valves), but has not previously been reported in brain abscesses [74].

16S NGS analysis offers a good supplement to other laboratory diagnostic methods, and the technology is well-suited for the detection of often culture-negative fastidious and anaerobic bacteria, which are common microbiological pathogens in the odontogenic brain abscess.

## Figures and Tables

**Table 1:** Reported cases of brain abscesses between 1981 and 2023 (and our findings) with *Capnocytophaga* spp., *Campylobacter gracilis* and *Arachnia propionica*, with sex, age, medical conditions/suggested anatomical location of infectious origin and location in the brain and co-occurrence of other microbiological pathogens of each case.

Cases Reported	Refs.	Number of Cases	Sex/Age (Years)	Dog/Cat Exposure	Medical Condition	Location of Brain Abscess	Bacterial Species	Other Microbial Pathogens
Archer 1985	[23]	1	M 39	Dog ownership		Frontal	Dysgonic fermenter 2 (DF-2)	
McNeil 1992	[24]	1	M 65		Diabetes	Frontoparietal	*Capnocytophaga* spp.	*Nocardia transvalensis*
Lozniewski 1999	[25]	1	M 39		Alcohol, dental, sinusitis	Frontoparietal	*Capnocytophaga* spp.	*Desulfovibrio *sp.*, Eubacterium exiguum, Streptococcus constellatus*
Engelhardt 2002	[26]	1			Congenital heart disease, dental		*Capnocytophaga* spp.	*Actinomyces* sp., *Streptococcus intermedius*
Sabbatani 2004	[27]	1	M adult	Facial cat bite		Frontal	*Capnocytophaga* spp.	
Wang 2007	[28]	1	M 7		Tooth extraction	Frontal	*Capnocytophaga ochracea*	
Ulivieri 2008	[29]	1	M 28	Dog bite		Temporal	*Capnocytophaga canimorsus*	
Ricciardi 2008	[30]	1	M 39		Congenital heart disease	Temporal	*Capnocytophaga* spp.	
Al Masalma 2009	[31]	1	M 61				*Capnocytophaga* spp.	*Campylobacter gracilis, Eikenella corrodens, Fusobacterium naviforme, Fusobacterium nucleatum, Parvimonas micra, Staphylococcus epidermidis, S. intermedius*
Kommedal 2014	[32]	4	M 69				*Capnocytophaga* spp.	
Kommedal 2014	[32]	4	M 58				*Capnocytophaga* sp. HOT-338	Actinomyces *sp.*, Aggregatibacter aphrophilus, C. gracilis *, Eikenella *sp. HOT-011*, F. nucleatum, Prevotella *sp. HOT-317*, S. intermedius
Kommedal 2014	[32]	4	M 60				*Capnocytophaga* spp.	*A. aphrophilus, C. gracilis, E. corrodens, Eubacterium brachy, Eubacterium yurii, F. nucleatum, P. micra, Prevotella *sp.*, Schaalia meyeri, S. intermedius, Tannerella forsythia*
Kommedal 2014	[32]	4	M 58				*Capnocytophaga* spp.	
Chen 2016	[33]	1	F 71	Dog ownership	TNF-α therapy	Cerebellar	*Capnocytophaga* spp.	
Senetar 2020	[34]	1	M 32		ASD	Multiple	*Capnocytophaga* spp.	*F. nucleatum*
Chesdachai 2021	[35]	1					*Capnocytophaga* spp.	
Westerström (this study)		1	M 59		Pulmonary AV fistula (HHT), dental	Parietooccipital	*Capnocytophaga ochracea*	*Arachnia propionica, C. gracilis, Candidatus Saccharibacteria* oral taxon 488, *Capnocytophaga *sp. HMT-323*, F. nucleatum, Johnsonella ignava, Schaalia georgiae, Treponema maltophilum, Treponema medium, Treponema socranskii, T. forsythia*
Johnson 1985	[36]	2			Sinusitis		*Campylobacter gracilis*	
Johnson 1985	[36]	2					*Campylobacter gracilis*	
Wolpow 1990	[37]	1	F 57		Pulmonary AV fistula (HHT)	Thalamic	*Campylobacter gracilis*	Bacteroides fragilis, Coccobacilliform organism **, Eubacterium *sp.*, F. nucleatum, P. micra
de Vries 2008	[38]	1	F 35		Post-partum		*Campylobacter gracilis*	*Streptococcus constellatus, Gram-positive cocci*
Al Masalma 2009	[31]	1	M 61				*Campylobacter gracilis*	*Capnocytophaga *sp.*, E. corrodens, F. naviforme,* *F. nucleatum, P. micra, Staphylococcus epidermidis, S. intermedius*
Hobson 2011	[39]	1	M 35		Tooth extraction, pregnancy		*Campylobacter gracilis*	*B. fragilis, Wolinella *spp.*, Prevotella buccae*
Kommedal 2011	[18]	2					*Campylobacter gracilis*	*F. nucleatum, Prevotella pleuritidis, S. meyeri, S. intermedius*
Kommedal 2011	[18]	2					*Campylobacter gracilis*	*Eikenella *sp.*, F. nucleatum, Peptostreptococcus *sp.*, S. meyeri*
Al Masalma 2012	[40]	1	M 35		Sinusitis		*Campylobacter gracilis*	F. nucleatum, P. micra, Proteobacteria ***, S. meyeri, S. epidermidis, S. intermedius
Kommedal 2014	[32]	8	M 44		Sinusitis, dental		*Campylobacter gracilis*	*A. aphrophilus, E. brachy, Fusobacterium *sp.*, S. georgiae, S. meyeri, P. micra, Parvimonas *sp.*, S. intermedius*
Kommedal 2014	[32]	8	F 51		Dental		*Campylobacter gracilis*	*A. aphrophilus, Campylobacter rectus, E. corrodens, F. nucleatum, Parvimonas *sp.*, S. meyeri*
Kommedal 2014	[32]	8	M 29		Dental		*Campylobacter gracilis*	*E. brachy, E. corrodens, E. yurii, F. nucleatum, Gemella morbillorum, P. micra, Prevotella oris, P. pleuritidis, S. meyeri, S. intermedius, T. forsythia*
Kommedal 2014	[32]	8	F 45		Oral mucositis, immunocompromised		*Campylobacter gracilis*	*E. corrodens, F. nucleatum, Fusobacterium *sp.*,* *P. micra, Prevotella *sp.*, S. meyeri, S. intermedius*
Kommedal 2014	[32]	8	M 64		Dental		*Campylobacter gracilis*	*A. aphrophilus, E. brachy, E. corrodens, Fusobacterium *sp.*, J. ignava, Parvimonas *sp.*, S. meyeri, S. intermedius*
Kommedal 2014	[32]	8	M 58				*Campylobacter gracilis* *	*Actinomyces *sp.*, A. aphrophilus, C. *sp. HOT-338*, Eikenella *sp. HOT-011*, F. nucleatum, Prevotella *sp. HOT-317*, S. intermedius*
Kommedal 2014	[32]	8	M 60				*Campylobacter gracilis*	*A. aphrophilus, Capnocytophaga *sp.*, E. brachy, E. corrodens, E. yurii, F. nucleatum, P. micra, Prevotella *sp.*, S. meyeri, S. intermedius, T. forsythia*
Kommedal 2014	[32]	8	M 65				*Campylobacter gracilis*	*E. corrodens, F. nucleatum, G. morbillorum, P. micra, Parvimonas *sp.*, S. meyeri, S. intermedius*
Jang 2021	[41]	1			Dental		*Campylobacter gracilis*	*F. nucleatum*
Hansen 2021	[42]	5	M 64				*Campylobacter gracilis*	*S. meyeri, F. nucleatum, S. intermedius, E. corrodens, P. micra*
Hansen 2021	[42]	5	M 60		Dental		*Campylobacter gracilis*	*E. corrodens, F. nucleatum, P. micra, S. meyeri*
Hansen 2021	[42]	5	F 54		Dental		*Campylobacter gracilis*	A. aphrophilus, Candida albicans, E. corrodens, F. nucleatum, Streptococcus anginosus group ****, P. micra, Porphyromonas endodontalis, Prevotella conceptionensis, S. meyeri, S. intermedius
Hansen 2021	[42]	5	M 61		Dental		*Campylobacter gracilis*	F. nucleatum, P. micra, S. meyeri, S. anginosus group ****, S. intermedius
Hansen 2021	[42]	5	M 54		Dental		*Campylobacter gracilis*	*Alloprevotella tannerae, Anaeroglobus geminatus, Atopobium rimae, Bulleidia extructa, Dialister invisus, Dialister pneumosintes, F. nucleatum, P. micra, P. oris*
Westerström (this study)		1	M 59		Pulmonary AV fistula (HHT), dental	Parietooccipital	*Campylobacter gracilis*	*A. propionica, Ca. Saccharibacteria* oral taxon 488, *C. ochracea, Capnocytophaga *sp. HMT-323*, F. nucleatum, J. ignava, T. maltophilum, T. medium, S. georgiae, T. forsythia, T. socranskii*
Riley 1981	[43]	1	M 32		Eisenmenger syndrome		*Arachnia propionica*	
Chau 2012	[44]	1	M 33		Eisenmenger syndrome		*Arachnia propionica*	
Westerström (this study)		1	M 59		Pulmonary AV fistula (HHT), dental	Parietooccipital	*Arachnia propionica*	*C. gracilis, Ca. Saccharibacteria* oral taxon 488, *C. ochracea, C. *sp.* *HMT-323*, F. nucleatum, J. ignava, T. maltophilum, T. medium, S. georgiae, T. forsythia, T. socranskii*

* culture ** species not determined *** detected at phylum level only **** culture *S. anginosus* group and 16S result with *S. intermedius*.

**Table 2:** Risk factors for the development of a polymicrobial anaerobic brain abscess with one of the pathogens *Capnocytophaga* spp., *C. gracilis and A. propionica* reported in published literature.

Risk	*Capnocytophaga* spp. *n* = 17	*Campylobacter gracilis n* = 22	*Arachnia propionica n* = 3
Dental	4	10	1
Exposure to dog or cat	3	-	-
Sinusitis	2	3	-
Congenital condition with right-to-left shunt	3	1	3
Immunocompromised condition	3	3	-

**Table 3:** Taxonomic assignment of bacterial species identified in brain abscess by next-generation sequencing of the 16S rRNA gene. Species identification was done according to CLSI guidelines; ≥99% homology with a high-quality reference for valid species identification and a minimum distance of >0.8% to the next alternative species.

Organism	% ID	Abundance	% Abundance
*Fusobacterium nucleatum complex*	100	62,045	23.3
*Arachnia propionica*	99.8	61,240	23
*Capnocytophaga* sp. HMT-323	99.5	54,700	20.6
*Campylobacter gracilis*	100	42,552	16
*Treponema medium*	99.3	27,373	10.3
*Treponema maltophilum*	100	12,158	4.6
*Schaalia georgiae*	99.1	3812	1.4
*Treponema socranskii*	99.8	1140	0.4
*Ca. Saccharibacteria* oral taxon 488	100	724	0.3
*Tannerella forsythia*	100	29	0
*Johnsonella ignava*	99	23	0

## Data Availability

The original contributions presented in this study are included in the article. Further inquiries can be directed to the corresponding author.

## List of Abbreviations

HHT	Hereditary Hemorrhagic Telangiectasia
PAVM	Pulmonary arteriovenous malformation
rRNA	ribosomal RNA; Ribonucleic Acid
MIC	Minimum Inhibitory Concentration
MALDI-TOF MS	Matrix-assisted laser desorption/ionization time-of-flight mass spectrometry
NGS	Next-generation sequencing
rDNA	ribosomal DNA; Deoxyribonucleic Acid
CLSI	Clinical & Laboratory Standards Institute
EUCAST	European Committee on Antimicrobial Susceptibility Testing
ECOFF	Epidemiological Cut-off value
TNF-α	Tumor-Necrosis-Factor alpha
